# The semantics of Chemical Markup Language (CML): dictionaries and conventions

**DOI:** 10.1186/1758-2946-3-43

**Published:** 2011-10-14

**Authors:** Peter Murray-Rust, Joe A Townsend, Sam E Adams, Weerapong Phadungsukanan, Jens Thomas

**Affiliations:** 1 Unilever Centre for Molecular Science Informatics, Department of Chemistry, Lensfield Road, Cambridge CB2 1EW, UK; 2 Department of Chemical Engineering, Pembroke Street, Cambridge CB2 3RA, UK; 3 STFC Daresbury Laboratory, Daresbury Science and Innovation Campus, Warrington WA4 4AD, UK

## Abstract

The semantic architecture of CML consists of conventions, dictionaries and units. The conventions conform to a top-level specification and each convention can constrain compliant documents through machine-processing (validation). Dictionaries conform to a dictionary specification which also imposes machine validation on the dictionaries. Each dictionary can also be used to validate data in a CML document, and provide human-readable descriptions. An additional set of conventions and dictionaries are used to support scientific units. All conventions, dictionaries and dictionary elements are identifiable and addressable through unique URIs.

## Introduction

From an early stage, Chemical Markup Language (CML) was designed so that it could accommodate an indefinitely large amount of chemical and related concepts. This objective has been achieved by developing a dictionary mechanism where many of the semantics are added not through hard-coded elements and attributes but by linking to semantic dictionaries. CML has a number of objects and object containers which are abstract and which can be used to represent the structure and datatype of objects. The meaning of these, both for humans and machines, is then realised by linking an appropriate element in a dictionary.

**Figure 1:**
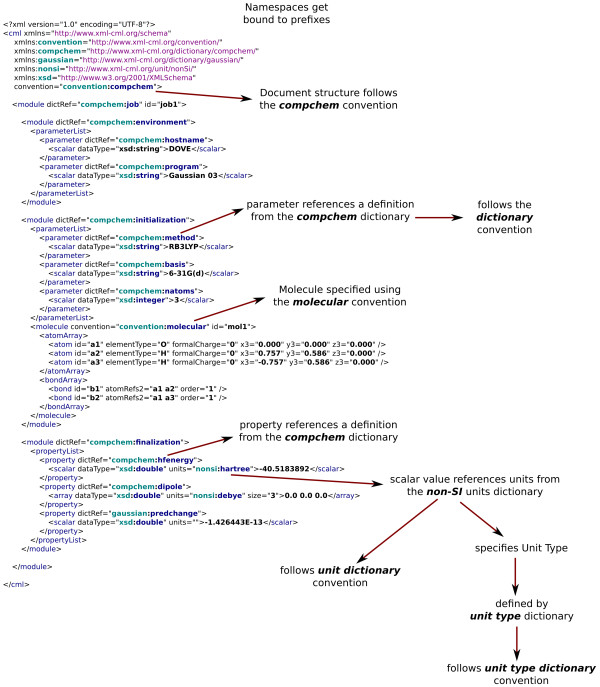
**The primary semantic components of CML**. Elements in a document link to conventions, dictionaries and units through attributes. The referenced resources are themselves constrained by specification documents (convention spec, dictionary spec, system of units) with unique URIs. Within the dictionaries and the unit collections, every entry has a unique ID and when combined with the dictionary URI produces a globally-unique identifier.

The dictionary approach was inspired by the CIF dictionaries [1] from the International Union of Crystallography (IUCr) and has a similar (in many places isomorphous) structure to that project. The design allows for an indefinitely large number of dictionaries created by communities within chemistry who recognise a common semantic approach and who are prepared to create the appropriate dictionaries. At an early stage, CML provided for this with the concept of "convention". This attribute is an indication that the current element and its descendants obey semantics defined by a group of scientists using a particularly unique label (Figure 1).

During the evolution of CML we explored a number of syntactic approaches to representing and imposing semantics through dictionaries. These have ranged from a formally controlled ontology (ChemAxiom [2]) which is consistent with OWL2.0 [3] and the biosciences' Open Biological and Biomedical Ontologies (OBO)[4] framework, to uncontrolled folksonomy-like tagging. Although we have implemented ChemAxiom and it is part of the bioscientists' description of chemistry, we regard it as too challenging for the current practice of chemistry and unnecessary for its communication. This is because chemistry has a well-understood (albeit implicit) ontology and the last 15 years have confirmed that it is highly stable. The power of declaration logic is therefore not required in building semantic structures. The consequence is that some of the mechanics of the semantics must be hard-coded, but this is a relatively small part and primarily consists of the linking mechanism and the treatment of scientific units of measurement. At the other end of the spectrum, we have found that the folksonomy approach is difficult to control without at least some formal semantic labelling. We have also found that there is considerable variation in how sub-communities approach their subject, and we do not wish to be prescriptive (even if we could). For example, the computational solids group (CMLComp) insisted that a molecule should not contain bonds as they did not exist, whereas the chemical informatics community is concerned not only that bonds should exist but that they should be annotated with their formal bond order.

**Figure 2:**
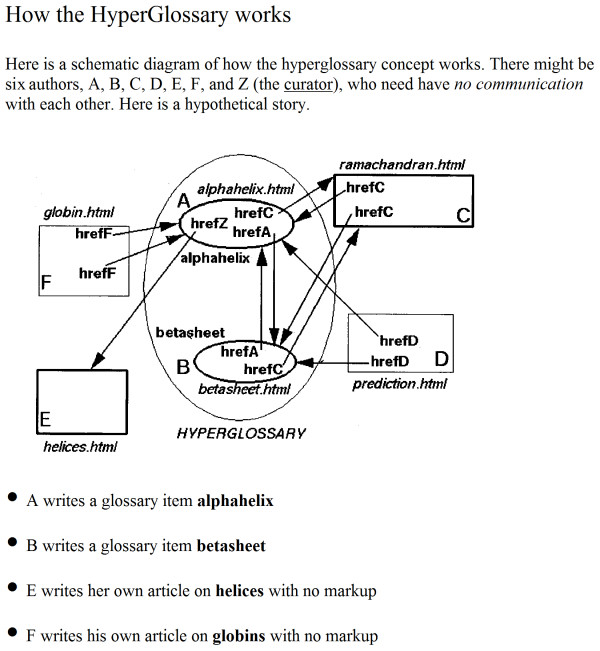
**The original design for CML semantic architecture (1996)**. This shows how different groups can create their own semantics and inter-operate. The concept has been proven over 15 years with appropriate changes to the terminology (*i.e*. we now talk of linked metadata rather than a hyperglossary).

The design of CML has always been based on the need for dictionaries, and has also recognised that there are different conventions within chemical practice. The original design (Figure 2) shows the linked dictionary concept and this has proved resilient and is the basis of the current architecture. However, the precise representation has varied over the years. This article represents a convergence and crystallisation of the semantic environment of CML, and we believe that there are now no immediate requirements for early refinement. This paper can therefore be used, we hope, for several years as a reference in a more robust manner than has been possible up to now. However, the exact practice of the CML community will be primarily governed by public discussions on mailing lists and formal releases of software and specifications.

This practice and principles are general to all the semantic elements in this article, and is best illustrated in the requirements for creating a convention and enforcing it. In the spirit of communal development, any sub-community is at liberty to create their own convention without formal permission from any central governance, subject to the requirement that it must be valid against the (very flexible) CML Schema 3 [5]. This is done by associating the convention with a unique namespace identifier and the convention specification shows how this must be done, but does not dictate the contents or scope of any convention. In this way, an indefinite number of sub-communities can develop and 'do their own thing' without breaking the CML semantics. The success of a convention is then a social, not technical, phenomenon. If group A develops a convention and groups B, C and D adopt it then there is wide interoperability. If A develops a convention and B develops an alternative then there is fragmentation. It's not always a bad thing to have "more than one way to do it"[6], but it can it make life very complex for software developers.

The price for this freedom is that a community cannot by default expect other users of CML to adopt their convention. If a community wishes its convention to be used, it needs to educate it in how CML can support it, and almost always to create or re-use software to support the convention. Thus, for example, the CMLSpect convention is supported by the JSpecView [7] software, which has a vigorous community of practice. Similarly, the CMLCryst convention (not yet released) is being driven by the development of the CrystalEye [8] knowledgebase and its adoption by the IUCr.

The dictionary reference mechanism (the dictRef attribute) was designed to have a namespace-oriented value; *i.e*. it has a prefix as well as a local name. Although this approach is not formally supported by XML, it is widespread in approaches such as XSD Schema. This has turned out to be a valuable design as it is isomorphic to the use of namespaced URIs and indeed the dictRef attribute can be automatically translated to and from the URI formulation. This means that CML is semantically compatible with the emergence of Linked Open Data (LOD) on the Open web, and that CML documents and dictionaries can be used in this with little more than syntactic conversion. In our own practice, we now enforce the discipline that dictRef values must be QNames[9] and that both the namespace and the local entry should be resolvable.

The role attribute has been used for a variety of purposes in the past but is now developed as a general "tagging" tool. A typical example is shown in the 'Roles' section below.

The semantic tools (dictionary, convention and role) have been fluid over the last decade and there are examples where their use is not compatible with this paper. However, the tools to support them will work with modern CML libraries.

The current tools in CML for adding semantics are therefore:

• **convention**. This represents a community of practice in chemistry and the attribute is used to label an element and its descendants which practice these semantics.

• **dictRef**. The formal mechanism of associating semantics with an abstract data object.

• **role**. An uncontrolled attribute which can be used in a folksonomy-like manner (microformats) and which has similarities to HTML's class attribute.

• **units****and ****unitType**. Attributes which allow scientific units of measurement to be added to numeric quantities in CML.

We now discuss each of these approaches in detail.

## Semantic Elements of CML

### Convention

The initial (1996) use of convention was limited to certain elements such as bond to represent the different values that different communities might use. It has now grown to be a key concept in defining communities of practice, having started to be used *ca*. 2005 when individuals and groups worked to create sub-domains of CML. The leading areas were reactions (mainly enzymes), spectroscopy, crystallography and computational chemistry (compchem). It emerged from these exercises that the elements and attributes of CML were sufficient to support the sub-community but that additional semantics in their use and constraints was necessary. Thus, for example, the CMLSpect [10] community decided that a spectrum must have a child representing the data in the spectrum (it is still possible to have an empty spectrum in CML but it would be used by a different community for a different purpose).

Conventions specify a minimal set of elements and document structure that a community has agreed to. Other elements may be included in a document, but may be transparently ignored by processing software.

Thus, a convention offers the following:

• an announcement that an identified community cares about a sub-domain of chemistry.

• a prose description of the scope and constraints and practice of the convention.

• a validator [11] that determines whether a given document conforms to a convention (and where it deviates).

In addition for software developers it offers:

• a statement as to what the components in a convention are, and how they can be combined.

• indications of what constraints may/must/should be imposed on CML documents valid against this convention.

• an indication or a guarantee as to what CML components may be found in a conformant document.

• an indication of their semantics.

CML Schema 3 is less restrictive than Schema 2.4 [12] and is designed to be used in conjunction with conventions. The loosening of the restrictions in the schema mean that it is schema-valid to create documents which do not make chemical sense (such as molecules being the children of atoms and bonds being defined in a molecule with no atoms present). The chemical validity and constraints are now imposed through the use of conventions and XSLT/XPath. @convention signifies that the element and its descendants must obey a convention, probably enforced by software and with defined semantics. There MUST (the keyword 'MUST' should be interpreted as described in RFC 2119, http://www.ietf.org/rfc/rfc2119.txt) be a convention document describing a convention.

Currently supported conventions (see Figure 1) are:

• dictionary (for which the namespace is http://www.xml-cml.org/convention/dictionary).

• molecular (namespace: http://www.xml-cml.org/convention/molecular).

• compchem (namespace: http://www.xml-cml.org/convention/compchem).

• unit-dictionary (namespace: http://www.xml-cml.org/convention/unit-dictionary).

• unitType-dictionary (namespace: http://www.xml-cml.org/convention/unitType-dictionary).

Examples of constraints implemented in the molecular convention are:

• an atomArray must have at least one atom child.

• the value of an atom's id must be unique within the eldest containing molecule.

• a bond element must have an atomRefs2 attribute.

• a bond must be between atoms within the same molecule.

### Dictionaries

In a similar way, a dictionary ecology [13] has developed supporting an extensible set of concepts in CML documents. The dictionaries add semantics to the CML primitives, particularly property and parameter. Thus, for example, a melting point is described by a property which is linked to a dictionary reference (dictRef). Therefore any concept which can be represented by the abstract CML elements can have additional semantics from a dictionary. Because the dictionary itself is semantic, it is possible to describe constraints and elaborations in the dictionary that can then be added to the document. For example, a dictionary can specify scientific units of measurement which would be the default for a reported property or parameter. Our current concept is that there are core dictionaries which are likely to be commonly used in many areas of chemistry. These include common physical properties (*e.g*. melting point) and common metadata such as users and dates. Conventions will almost certainly have one or more dictionaries so that compchem has an extended dictionary of concepts such as convergent limits, energies, gradients and so forth. The MACiE [14] dictionary used the IUPAC Gold Book [15] to define terms in reactions and the Atmospheric Chemistry dictionary is again taken from IUPAC [16].

One important way of creating dictionaries is to extract terms and discourse from CML documents. A particular example is the markup of concepts created in computational chemistry and here we often associate a given program or code with a dictionary specific to that program/code. Thus, for example, a program/code might use a set of keywords found nowhere else; currently around six such dictionaries exist, and the number is increasing. In these cases we often find the need for a hierarchy so that a code might use code-specific dictionary terms in addition to those in the general computational chemistry dictionary. Different programs sometimes produce data with the same label but a different interpretation; does "*density" *mean electron density or mass density? There can be any number of dictionaries (and we envisage one for each code, or ideally fewer). Each dictionary has a unique namespace so there are no collisions. The entries can be minimal (id, term, definition, *etc*.) but will usually indicate the data structure (scalar, array etc.), data type, constraints etc. The descriptions can be HTML and include all sorts of additional material (including SVG).

Applying @dictRef to an element asserts that it is defined in some way by a dictionary entry but does not generally transmit to descendants of that element. Thus:

< property @dictRef = 'foo:cpuInfo' > ... </property >

might specify that this property must be interpreted with the help of the cpuInfo entry in the foo dictionary. The @dictRef construct is most generally used for primitive types (scalar, array or matrix) though we are starting to see its use for compound types (*e.g*. parameter constraining a property). There MUST (the keyword 'MUST' should be interpreted as described in RFC 2119, http://www.ietf.org/rfc/rfc2119.txt) be a dictionary entry for a dictRef.

Example (from http://www.xml-cml.org/convention/dictionary):

< ?xml version = "1.0" encoding = "UTF-8" ? >

< dictionary xmlns = "http://www.xml-cml.org/schema"

xmlns:convention = "http://www.xml-cml.org/convention/"

xmlns:unit = "http://www.xml-cml.org/unit/nonSi/"

xmlns:unitType = "http://www.xml-cml.org/unit/unitType/"

xmlns:xhtml = "http://www.w3.org/1999/xhtml"

xmlns:xsd = "http://www.w3.org/2001/XMLSchema"

convention = "convention:dictionary"

title = "fundamental chemistry concepts"

namespace = "http://www.xml-cml.org/dictionary/dummy/"

dictionaryPrefix = "dummy">

<description>

<xhtml:p> This is an example dictionary

</xhtml:p>

</description>

<entry id = "molecmass" term = "Molecular Mass" dataType = "xsd:double" unitType = "unitType:amount" units = "unit:dalton">

<definition>

<xhtml:p>

The mass of one mole of a substance in unified atomic mass units (Dalton).

</xhtml:p>

</definition>

<description>

<xhtml:p>

The molecular mass (m) of a substance is the mass of one molecule of that substance, in unified atomic mass unit(s) u (equal to 1/12 the mass of one atom of the isotope carbon-12). This is numerically equivalent to the relative molecular mass (Mr) of a molecule, frequently referred to by the term molecular weight, which is the ratio of the mass of that molecule to 1/12 of the mass of carbon-12 and is a dimensionless number. Thus, it is incorrect to express relative molecular mass (molecular weight) in daltons (Da). Unfortunately, the terms molecular weight and molecular mass have been confused on numerous websites, which often state that molecular weight was used in the past as another term for molecular mass.

</xhtml:p>

<xhtml:p>

Molecular mass differs from more common measurements of the mass of chemicals, such as molar mass, by taking into account the isotopic composition of a molecule rather than the average isotopic distribution of many molecules. As a result, molecular mass is a more precise number than molar mass; however it is more accurate to use molar mass on bulk samples. This means that molar mass is appropriate most of the time except when dealing with single molecules.

</xhtml:p>

</description>

</entry>

<entry id = "molarmass" term = "Molar Mass" dataType = "xsd:double" unitType = "unitType:amount" units = "unit:dalton">

<definition>

<xhtml:p>

The mass per amount of substance.

</xhtml:p>

</definition>

<description>

<xhtml:p>

Molar mass, symbol M, is a physical property characteristic of a given substance (chemical element or chemical compound), namely its mass per amount of substance. The base SI unit for mass is the kilogram and that for amount of substance is the mole. Thus, the derived unit for molar mass is kg/mol. However, for both practical and historical reasons, molar masses are almost always quoted in grams per mole (g/mol or g mol-1), especially in chemistry.

</xhtml:p>

<xhtml:p>

Molar mass is closely related to the relative molar mass (Mr) of a compound, the older term formula weight and to the standard atomic masses of its constituent elements. However, it should be distinguished from the molecular mass (also known as molecular weight), which is the mass of one molecule (of any single isotopic composition) and is not directly related to the atomic mass, the mass of one atom (of any single isotope). The dalton, symbol Da, is also sometimes used as a unit of molar mass, especially in biochemistry, with the definition 1 Da = 1 g/mol, despite the fact that it is strictly a unit of molecular mass (1 Da = 1.660 538 782(83)×10-27 kg).

</xhtml:p>

</description>

</entry>

</dictionary>

### Roles

A third approach to semantics is driven by the need to 'tag' information, and for this we provide the role attribute. Roles are less formalised than dictRef or convention in that they do not (at this time) need to refer to a formal specification, and are therefore available for folksonomies and human-readable *ad hoc *semantics. They may, of course, link to formal semantic documents if required, though this cannot be enforced except by convention.

@role signifies how an element is to be interpreted. In some CML architectures, @role might be used as a human-readable tag - *i.e*. part of a folksonomy, while in other cases @convention could be used as a machine-readable tag and impose machine semantics. There are currently no constrained semantics or vocabulary for @role.

Example showing how role is used in the definition of a fragment within a polymer [17]:

<?xml version = "1.0" encoding = "UTF-8"?>

<fragment id = "cl_nsp2_methyl" convention = "cml:PML-complete" xmlns = "http://www.xml-cml.org/schema" xmlns:g = "http://www.xml-cml.org/mols/geom1">

<molecule role = "fragment" id = "benzene_1">

<atomArray>

<atom elementType = "C" x3 = "9.526706134000763" y3 = "3.869733600000001" z3 = "5.213518402229052" id = "benzene_1_a1">

<label dictRef = "cml:torsionEnd"> r6 </label>

</atom>

<atom elementType = "C" x3 = "10.243299413197152" y3 = "3.932398500000001" z3 = "6.439022942911609" id = "benzene_1_a2">

<label dictRef = "cml:torsionEnd"> r1 </label>

</atom>

<atom elementType = "C" x3 = "8.713504556428543" y3 = "2.7185301000000006" z3 = "5.01720505576243" id = "benzene_1_a6">

<label dictRef = "cml:torsionEnd"> r5 </label>

</atom>

<atom elementType = "R" x3 = "8.385888936961882" y3 = "2.655387420737078" z3 = "4.323244676535362" id = "benzene_1_r6"/>

<atom elementType = "C" x3 = "10.119474056141831" y3 = "2.9008920000000007" z3 = "7.3834992125284815" id = "benzene_1_a3">

<label dictRef = "cml:torsionEnd"> r2 </label>

<label dictRef = "cml:torsionEnd"> r2 </label>

<label dictRef = "cml:torsionEnd"> r3 </label>

</atom>

<atom elementType = "C" x3 = "9.320371405363035" y3 = "1.8151698000000005" z3 = "7.151684115065878" id = "benzene_1_a4">

<label dictRef = "cml:torsionEnd"> r3 </label>

</atom>

<atom elementType = "R" x3 = "9.280916015724046" y3 = "1.2657016684721403" z3 = "7.6896692864820775" id = "benzene_1_r4"/>

<atom elementType = "C" x3 = "8.610030693701125" y3 = "1.7243409000000007" z3 = "5.934289686115539" id = "benzene_1_a5">

<label dictRef = "cml:torsionEnd"> r4 </label>

</atom>

<atom elementType = "R" x3 = "10.697234803620145" y3 = "4.543958438540135" z3 = "6.552323882423661" id = "benzene_1_r2"/>

<atom elementType = "Cl" formalCharge = "0" hydrogenCount = "0" id = "cl_2_a1" x3 = "9.692011995771473" y3 = "5.151468187879777" z3 = "4.018767207085518"/>

<atom elementType = "N" formalCharge = "0" hydrogenCount = "0" id = "nsp2_3_n1" x3 = "10.889175042006798" y3 = "2.9930090553818314" z3 = "8.690968080404991"/>

<atom elementType = "R" formalCharge = "0" hydrogenCount = "0" id = "nsp2_3_r3" x3 = "10.937618525919527" y3 = "3.6140787328234207" z3 = "9.108611091395234"> </atom>

<atom elementType = "R" formalCharge = "0" hydrogenCount = "0" id = "nsp2_3_r2" x3 = "11.21097670608745" y3 = "2.3333885683637092" z3 = "8.845384731388283">

<label dictRef = "cml:torsionEnd"> r1 </label>

</atom>

<atom elementType = "C" id = "me_4_a1" x3 = "7.720415546204135" y3 = "0.5004327093517826" z3 = "5.6475256189660525"/>

<atom elementType = "H" id = "me_4_a6" x3 = "7.8973212132921615" y3 = "0.15287314794224827" z3 = "4.629221766363621">

<label dictRef = "cml:torsionEnd"> r1 </label>

</atom>

<atom elementType = "H" id = "me_4_a7" x3 = "7.962448186970064" y3 = "-0.2976542125451189" z3 = "6.350030754819878"/>

<atom elementType = "H" id = "me_4_a8" x3 = "6.673285676722817" y3 = "0.7788619666709824" z3 = "5.760051994135236"/>

</atomArray>

<bondArray>

<bond order = "2" id = "benzene_1_a1_benzene_1_a2" atomRefs2 = "benzene_1_a1 benzene_1_a2"/>

<bond order = "1" id = "benzene_1_a1_benzene_1_a6" atomRefs2 = "benzene_1_a1 benzene_1_a6"/>

<bond order = "1" id = "benzene_1_a3_benzene_1_a2" atomRefs2 = "benzene_1_a3 benzene_1_a2"/>

<bond order = "1" id = "benzene_1_a2_benzene_1_r2" atomRefs2 = "benzene_1_a2 benzene_1_r2"/>

<bond order = "1" id = "benzene_1_r6_benzene_1_a6" atomRefs2 = "benzene_1_r6 benzene_1_a6"/>

<bond order = "2" id = "benzene_1_a5_benzene_1_a6" atomRefs2 = "benzene_1_a5 benzene_1_a6"/>

<bond order = "2" id = "benzene_1_a3_benzene_1_a4" atomRefs2 = "benzene_1_a3 benzene_1_a4"/>

<bond order = "1" id = "benzene_1_a5_benzene_1_a4" atomRefs2 = "benzene_1_a5 benzene_1_a4"/>

<bond order = "1" id = "benzene_1_a4_benzene_1_r4" atomRefs2 = "benzene_1_a4 benzene_1_r4"/>

<bond atomRefs2 = "benzene_1_a1 cl_2_a1" order = "S" id = "benzene_1_a1_cl_2_a1"/>

<bond order = "S" atomRefs2 = "nsp2_3_n1 nsp2_3_r2" id = "nsp2_3_n1_nsp2_3_r2"/>

<bond order = "S" atomRefs2 = "nsp2_3_n1 nsp2_3_r3" id = "nsp2_3_n1_nsp2_3_r3"/>

<bond atomRefs2 = "benzene_1_a3 nsp2_3_n1" order = "S" id = "benzene_1_a3_nsp2_3_n1"/>

<bond order = "1" atomRefs2 = "me_4_a1 me_4_a6" id = "me_4_a1_me_4_a6"/>

<bond order = "1" atomRefs2 = "me_4_a1 me_4_a7" id = "me_4_a1_me_4_a7"/>

<bond order = "1" atomRefs2 = "me_4_a1 me_4_a8" id = "me_4_a1_me_4_a8"/>

<bond atomRefs2 = "benzene_1_a5 me_4_a1" order = "S" id = "benzene_1_a5_me_4_a1"/>

</bondArray>

</molecule>

</fragment>

### Units

The final component of the semantic framework is scientific units of measurement. In these we specify the type of the unit (unitType), which itself has a specific dictionary [18]. Every units attribute therefore has a unitType and the units are described in their own dictionaries where we expect a variety of approaches. Dictionaries of CGS (centimetre gram second) units, atomic units and even units connected with a particular (compchem) code may all be encountered.

These "essentials" are adapted from NIST Special Publication 811 (SP 811)[19] and NIST Special Publication 330 (SP 330)[20]. We use the terminology from NIST, with some variation, and quote verbatim to avoid confusion:

"A **quantity in the general sense **is a property ascribed to phenomena, bodies, or substances that can be quantified for, or assigned to, a particular phenomenon, body, or substance. Examples are mass and electric charge."

CML uses the term "unitType" to describe this concept (in part to avoid confusion with the next definition). This also shows the strong computational relationship between unit and its type. We believe that essentially all uses of "unitType" map onto quantity.

"A **quantity in the particular sense **is a quantifiable or assignable property ascribed to a particular phenomenon, body, or substance. Examples are the mass of the moon and the electric charge of the proton."

CML does not currently use this concept explicitly. Quantities are usually either parameters or properties (but not all parameters and properties (*e.g*. string values) map to quantities).

"A **physical quantity **is a quantity that can be used in the mathematical equations of science and technology."

CML honours this concept in that unitTypes can be associated with equations though this is complex and not yet widespread.

"A unit is a particular physical quantity, defined and adopted by convention, with which other particular quantities of the same kind are compared to express their value."

CML maps onto this concept through the units attribute and dictionary.

"The **value of a physical quantity **is the quantitative expression of a particular physical quantity as the product of a number and a unit, the number being its numerical value. Thus, the numerical value of a particular physical quantity depends on the unit in which it is expressed."

CML supports this in the scalar, array and matrix elements which, if numeric, should be supported by a units attribute.

CML will honour specifications of units and unitTypes created by authorities such as NIST as they should rightly be the creators and disseminators. A UnitsML [21] has been many years in incubation but now seems to be close to production release. CML will continue to use its own semantics for units but may also include interoperability with NIST.

The CML system of units goes somewhat beyond NIST in that it is not limited to physical science and has to support concepts such as mg (drug)/kg (animal) where the semantics of the experiment have to be linked (this is not a simple dimensionless number - "drug" and "animal" do not cancel). CML units allow for dimensions and other concepts to be associated with "dimensionless", such as ppm). CML software (JUMBO [22]) allows for the values and units to be recomputed ("unit conversion") and for simple dimensional analysis. Entries in unitType dictionaries conforming to the unitType dictionary convention must specify dimensions.

Users can create their own unitTypes and units as long as these conform to the CML conventions. There are many biological units (*e.g*. "The optimum dose of rIL2 was 100-500 units (Jurkat units)/ml, "[23]) which do not fit easily into the seven primary SI concepts, but are still critical attributes of the experiment. The general structure of the dictionaries is likely to be:

• A single, community-driven and maintained dictionary for unitTypes. Since there are infinitely many of these (*e.g*. fifth virial coefficient units), we see this being gradually and carefully extended.

• A number of local unitTypes (*e.g*. Jurkats).

• A single dictionary for SI units[24] (paralleling the unitTypes).

• A small number of core dictionaries for units in different non-SI systems [25] (*e.g*. CGS, atomic units, *etc*.).

• A larger number of convention-specific units dictionaries.

## Creating dictionaries

The biosciences have several approaches for creating ontologies, such as the Gene Ontology (GO)[26]. GO was designed as a thesaurus to which individuals and groups could contribute. It has a directed acyclic graph (DAG) structure, where an entry can have several parents and several children. The hierarchy honours the broader/narrower term approach and used three axes (cellular component, molecular function, biological process) but is designed primarily for human navigability rather than machine computability. It and other dictionaries have been transformed to fuller OWL-compliant ontologies using the file format guide provided [27].

We use the following approaches for creating dictionaries:

**• Borrow from established dictionaries **(IUPAC, IUCr, Wikipedia) and convert to CML. The main challenge is that many of the terms are broad concepts and follow human rather than machine conventions. This approach was used for the MaCiE dictionary with terms borrowed from IUPAC where possible and with a hierarchy expressed in CML. We have also translated the IUCr's CIF dictionary into CML format [28], and this is used in, for example, the CrystalEye system.

**• Observe and collect discourse/practice**, both in program input/output and formulaic text. We create or collect a corpus of documents and extract the common terms. Assuming that they are associated with cml:property or cml:parameter they will require a dictRef. The target of this dictRef is an entry in a dictionary and the first task is to determine which dictionary is most appropriate.

These processes lead to a community of dictionaries, with an implied but not necessarily explicit hierarchy.

## Detailed use cases of dictionary construction

•With the ChemicalTagger [29] system, we have built a natural language framework which recognises parts of speech and phrase. With over 100, 000 patents analysed we have a large corpus representing the current usage in describing chemical synthesis. The automatic analysis [30] of this corpus throws up a variety of abstractions common to many of the texts, in particular for the actions and methods used to describe chemical syntheses. Currently we have extracted 21 types of action phrase from this corpus:

Add, ApparatusAction, Concentrate, Cool, Degass, Dissolve, Dry, Extract, Filter, Heat, Partition, Precipitate, Purify, Quench, Recover, Remove, Stir, Synthesize, Wait, Wash and Yield.

Coupled with these phrases are qualifiers (sometimes English language adverbs) and specific uses of nouns which can be additionally used to label a text. This is an example of a small natural language driven dictionary into which a large number of specific terms can be entered.

•In the Quixote project [31,32] we are creating a semantic infrastructure for compchem. Unlike crystallography, where the community has for many years sat in real and virtual committee to decide on dictionaries and their contents, compchem has very little common practice in this area. There is no commonality of approach to labelling either the input or output of compchem calculations. Our belief is that there is a strong implicit similarity, even isomorphism, between the main computational codes, and that by analysing the discourse (*i.e*. the logfiles), we can collect and systematise the types of object referenced in the logfiles. To do this, we have taken a number of codes (Gaussian [33] (various versions), GAMESS-UK [34], Jaguar [35], NWChem [36], Quantum ESPRESSO [37]) and analysed much of their logfile structure and vocabulary. Although the level of detail varies between programs, there are somewhere between 100-500 concepts in total which can be precisely labelled and which could contribute to a communal dictionary. We are in the process of building a table (spreadsheet) of the terms which occur in codes and their occurrence (or absence) in each code. These normally occur as CML parameters. The concepts currently cover the following areas:

■Environment of the calculation. This includes machine configurations, version of code, time constraints, human and institutional metadata and other control parameters.

■The method of calculation *e.g*. the functional.

■The basis set or pseudo-potential.

■Any physical constraints imposed on the system (*e.g*. pressure, temperature or electric field).

■Levels of accuracy or cut-off desired in the calculation.

■Strategy of calculation and algorithms used (*e.g*. search for a transition state, reaction coordinates, frequencies *etc*.).

The output files normally deal with outcomes of running the job (*e.g*. abnormal termination, level of convergence achieved, elapsed time) and calculated properties.

**Figure 3:**
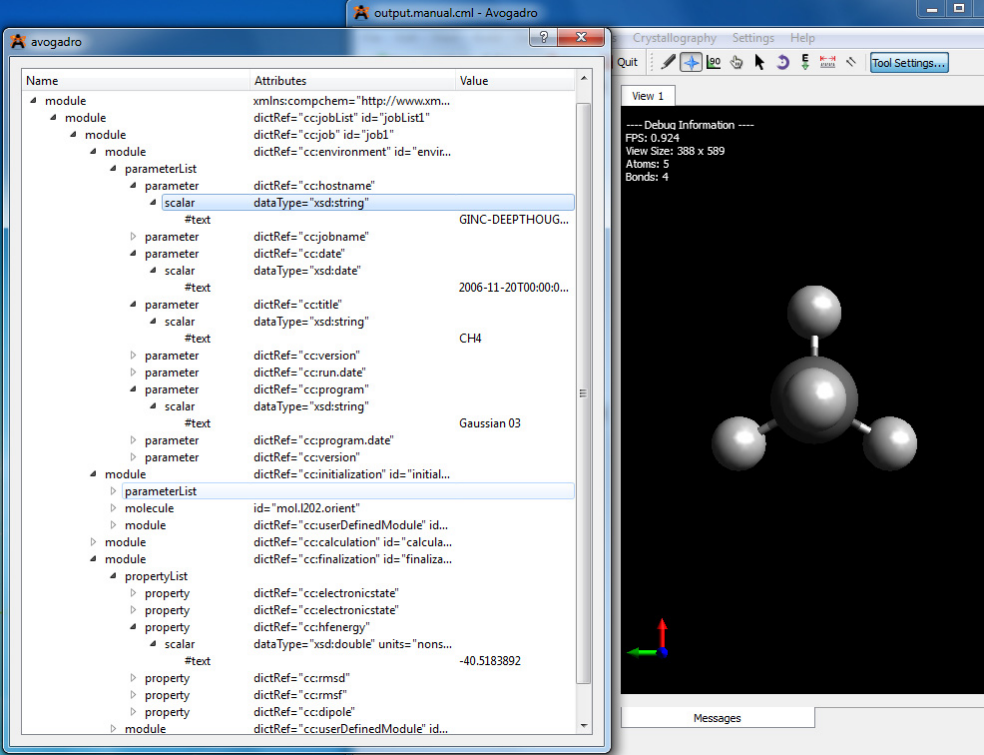
**A compchem-compliant document read into the Avogadro **[39]**browser and computational chemistry manager**. The structure of the document is shown with the primary subdivisions. Each piece of information is in a precisely specified position in the hierarchy, so that it may easily be discovered by processing software. For example the hostname must occur as a scalar child of parameter with a specific @dictRef, and so on.

Most of these concepts are common to all codes and where possible we are creating entries in a single common compchem dictionary [38] (Figure 3). In some cases, however, methods and properties are unique to one code, and many of the intricate details in the logfiles are not directly transferable. For that reason, we are using a hierarchy of dictionaries with the following components:

1. A dictionary common to all or most of computational chemistry (compchem dictionary).

2. A series of dictionaries, one per code, which is initially used to collect defined quantities in the output. At regular stages the community will decide whether these map onto concepts in the main compchem dictionary, and, in those cases, transfer their usage to that dictionary.

## Software support for dictionaries and units

Besides the markup support for dictionaries and units, they are only really useful in chemistry if they are supported by a software system. Some of this can be provided by Web 2.0 tools such as RDF which can be used to lookup whether referenced units are present in appropriate dictionaries. However, it is often important to carry out manipulations on units such as conversion between different systems and multiplier prefixes. For that reason we have developed a suite of software within the JUMBO system for these manipulations.

The following elements are established in CML:

• Dictionary.

• Entry.

• Unit type (and unit type list.)

• Unit (in unit list).

In our recent work with dictionaries (especially in computational chemistry) we use the entries to provide some of the semantics to be applied at "run-time". For example, a dictionary entry may define a syntactic template for the concept, or an enumeration of allowed values. In using the CIF dictionary, the data type (XSD: string, XSD: double) is used to enforce the type of the quantity being interpreted. Similarly, the enumeration of types of basis set can be used to check input and to expand the values. Any scientific discipline which wishes to use dictionaries and input units should find that our software design and implementation in JUMBO can be readily understood and may be appropriate for their domain.

## Conclusion

The use of conventions and dictionaries has proved of enormous value in the development and robustification of CML. With well-defined protocols, groups can take the formal specifications and build their own systems such that they not only do what they want, but do not break other CML software. We are currently working actively on computational chemistry and, with a wide range of different codes and types of problem, we expect to be able to show that the current architecture is capable of supporting these.

Assuming that semantic computational chemistry becomes widespread, the dictionaries will act as a catalyst to those communities to add more terms and to revise the precise usage of the concepts. It will also act as a demonstration to other areas of chemistry of the value of the convention/dictionary approach.

## Competing interests

The authors declare that they have no competing interests.

## Authors' contributions

PMR designed dictionaries and their schemas, and wrote the manuscript. JAT implemented conventions and the validator, and wrote the manuscript. SEA built supporting software, and wrote the manuscript. WP implemented conventions and dictionaries. JT designed compchem dictionaries. All authors have read and approved the final manuscript.
